# Implications of Gut Microbiota in Epithelial–Mesenchymal Transition and Cancer Progression: A Concise Review

**DOI:** 10.3390/cancers14122964

**Published:** 2022-06-16

**Authors:** Ishita Gupta, Shona Pedersen, Semir Vranic, Ala-Eddin Al Moustafa

**Affiliations:** 1 College of Medicine, QU Health, Qatar University, Doha P.O. Box 2713, Qatar; spedersen@qu.edu.qa (S.P.); svranic@qu.edu.qa (S.V.); 2 Biomedical Research Center, Qatar University, Doha P.O. Box 2713, Qatar

**Keywords:** microbiota, gut, epithelial–mesenchymal transition, dysbiosis, cancer

## Abstract

**Simple Summary:**

Recently, the interactions between microbiota and the host have been reported to induce the onset and progression of human cancer via epithelial–mesenchymal transition (EMT). In contrast, some microorganisms can protect against cancer growth, indicating an anticancer therapeutic action of such microbiota. In the review, we summarize findings from the literature, exploring the underlying mechanisms by which pathogenic microorganisms induce EMT. We also highlight the potential of exploiting these complex interactions for developing new biological therapies.

**Abstract:**

Advancement in the development of molecular sequencing platforms has identified infectious bacteria or viruses that trigger the dysregulation of a set of genes inducing the epithelial–mesenchymal transition (EMT) event. EMT is essential for embryogenesis, wound repair, and organ development; meanwhile, during carcinogenesis, initiation of the EMT can promote cancer progression and metastasis. Recent studies have reported that interactions between the host and dysbiotic microbiota in different tissues and organs, such as the oral and nasal cavities, esophagus, stomach, gut, skin, and the reproductive tract, may provoke EMT. On the other hand, it is revealed that certain microorganisms display a protective role against cancer growth, indicative of possible therapeutic function. In this review, we summarize recent findings elucidating the underlying mechanisms of pathogenic microorganisms, especially the microbiota, in eliciting crucial regulator genes that induce EMT. Such an approach may help explain cancer progression and pave the way for developing novel preventive and therapeutic strategies.

## 1. Introduction

Epithelial cells are apicobasal polarized cells that function as physical barriers. They are tightly bound to adjacent cells, and the extracellular matrix (ECM) is regulated by E-cadherins and cytokeratins, respectively [1,2]. However, under certain conditions, including developmental processes, wound healing, repair, and tumor progression, epithelial cells lose their high degree of plasticity and attain migratory and invasive capabilities [3]. During the alteration of epithelial cells, junctional proteins are relocalized, or a more severe event occurs, such as the epithelial–mesenchymal transition (EMT) initiation [4,5]. During EMT, epithelial cells undergo loss of cell-to-cell junction and reorganization of the actin cytoskeleton; thus, nonmotile epithelial cells are converted to motile and invasive mesenchymal phenotypic cells [6]. Morphologically, epithelial cells lose their polygonal phenotype and acquire an elongated fibroblast morphology; these events are regulated by vimentin, fibronectin, and N-cadherin [4,5]. EMT is characterized by loss of E-cadherin and translocation of β-catenin from the cell membrane to the nucleus, followed by activation of several mesenchymal markers (e.g., vimentin, fibronectin, and N-cadherin) [4,5].

Though several growth factors activate signaling pathways to control EMT gene expression, some EMT-signaling pathways are regulated by microbial pathogens [7,8]. 

Previous studies reported that microbe invasion might alter the transforming growth factor β (TGFβ); thus, the TGFβ receptor phosphorylates and activates transcription factors Smad-2 and Smad-3, which heterodimerize with Smad-4 to form the Smad complex [9,10,11]. The Smad complex recruits the Ras-MAPK pathway leading to cell growth; proliferation; differentiation; migration; and, therefore, cancer progression [12,13].

In this review, we present a brief overview of the human gut microbiome, focusing on gut dysbiosis during EMT. We present data from the literature that shed light on their possible role in this crucial event, further triggering carcinogenesis and its progression.

## 2. Microbiota

Of the total human cell count, around 90% are associated with the presence of microbiota, while the remaining 10% are microbiome-free [14]. Nevertheless, it is postulated that the number of microbial genes is approximately ten times higher than the number of human genes [14]. Primarily located in the gut, the microbes play a vital role in nutrient uptake [15] and influence the development of healthy intestinal immune responses [16]. Any modification or change in the microbiota composition disrupts the microbe–immune system relationship, further inducing the onset and development of several human inflammatory disorders that may lead to EMT [17,18].

The Human Microbiome Project (HMP) was a two-phase research initiative that used metagenomics and whole-genome sequencing in the first phase to recognize and distinguish the whole human microbiota [19]. In the second phase, the project revealed the role of microbes in human diseases using multiple omics techniques [19,20]. Although alterations in genes regulating DNA repair are mainly responsible for the onset and progression of tumorigenesis, the HMP indicated a role of dysbiotic microbiota in cancer progression [19]. With recent advancements, the use of genomics, epigenomics, proteomics, metabolomics, and transcriptomics elucidated host–microbiota interactions and their underlying mechanisms in human diseases; however, its role in carcinogenesis is still nascent. While viruses express active oncoproteins that can induce cell transformation leading to tumor formation or progression, dysbiosis-induced carcinogenesis arises after multiple hits [21]. An in vivo study using gnotobiotic (including germ-free) mouse models reported that microbes affect metabolism and inflammation, provoking the onset and progression of cancer [22].

Due to the extensive presence of microbes in the gut, several studies have primarily focused on the effects of altered microbiota in colorectal cancer pathogenesis [23,24,25,26,27,28,29,30,31,32]. Nevertheless, recent investigations have shown a correlation between dysbiosis and other cancers, including breast, oral, lung, skin, and reproductive tract [33,34,35,36,37,38,39,40,41]. The following section discusses the interplay between the host and the microbiota in triggering the onset of cancer via EMT. 

## 3. Microbiota-Induced Epithelial–Mesenchymal Transition

Microbes induce EMT by attaching to the mucosal layers and trigger the breakdown of intercellular adhesion between epithelial cells. Bacterial adhesins bind to epithelial proteins’ E-cadherin/catenin complex, thus altering cell polarity and downstream signaling pathways, leading to EMT [42]. A study by Chen and colleagues reported that immunosuppression due to severe inflammation that overwhelms both regulatory T-cells and dendritic cells was significantly associated with the onset of EMT [43,44]. In the colon, *Fusobacterium nucleatum* (*F. nucleatum*) enhances the release of inflammatory cytokines [45]; in the urogenital tract, infection with *Lactobacillus* spp. triggers the release of interleukins [39].

**Figure 1:**
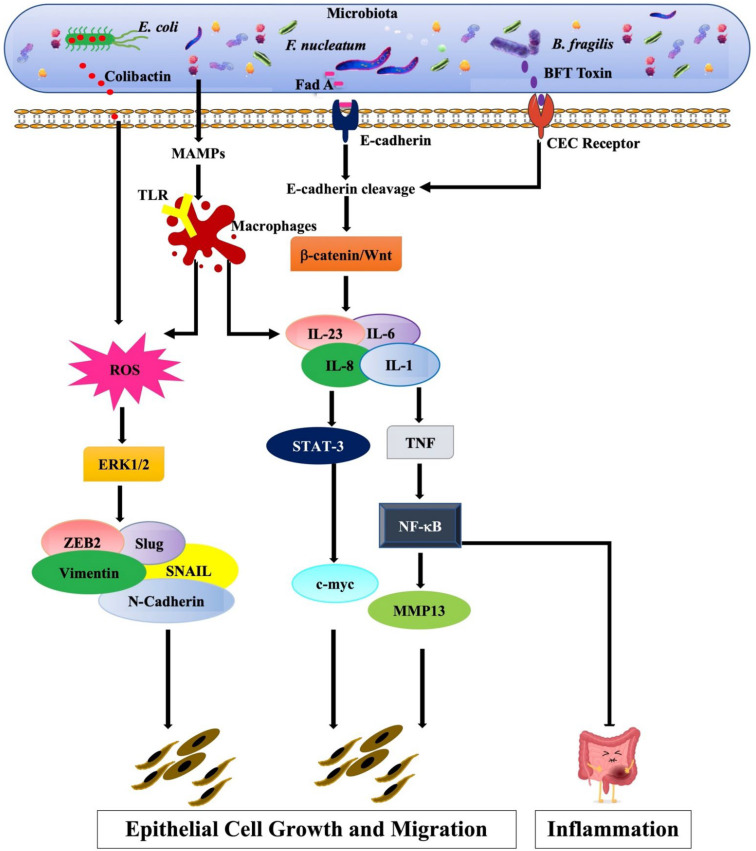
Molecular pathways depicting the microbiome-induced EMT and chronic inflammation. *F. nucleatum*: *E. coli* strains producing genotoxic compound colibactin can bind to the DNA leading to DNA damage by triggering reactive oxygen species (ROS) and activating the Erk pathway. Activation of Erk stimulates Vimentin and N-cadherin expression, leading to EMT. Microbes express microorganism-associated molecular patterns (MAMPs) and are recognized by macrophages via TLRs. They can either produce ROS from macrophages or trigger the production of proinflammatory cytokines (IL-1, IL-6, IL-8, IL-23, and TNF) via various signaling pathways. Proinflammatory cytokines can activate STAT3 and NF-κB signaling, leading to activation of c-myc oncogene and MMP13, respectively, which progress to EMT, chronic inflammation, and eventually cancer. Simultaneously, virulence factors, FadA and BFT, can disrupt E-cadherin and trigger β-catenin/Wnt signaling pathways resulting in subsequent activation of the STAT3 and NF-κB pathways.

One of the most common bacteria the Gram-negative, microaerophilic bacteria, *Helicobacter pylori* (*H. pylori*) is present in the digestive tract in approximately 50% of the population worldwide [46,47]. In addition to its causative role in inflammation and ulceration in gastric epithelial cells, *H. pylori* can trigger toll-like receptors-2 and -5 (TLR2 and TLR5) to activate NFκB [48]. On the other hand, the virulent cytotoxic factors of *H. pylori*, CagA, and VacA can disrupt epithelial cell function. CagA disrupts the apical junctional complex and actin-cytoskeletal rearrangements. In contrast, VacA destroys the barrier function of tight junctions, leading to loss of epithelial cell-to-cell adhesion and loss of cell polarity [49,50,51]. Brandt and colleagues [52] reported that CagA could induce the release of IL-8 via the Ras/Mek/Erk/NFκB signaling pathways (Figure 1). Following this, another study by Yin et al. [53] showed that pathogenic strains of *H. pylori* enhance the expression of vimentin, Snail, and Slug supported by upregulated levels of gastrin; MMP7; and soluble heparin-binding epidermal growth factor. The studies support the role of *H. pylori* in the remodeling of actin filaments leading to the onset of EMT [52,53].

Moreover, *E. coli* is present in the gastrointestinal tract within a few hours after birth and generally harmonizes with its human hosts [54]. However, during the loss of intestinal barrier permeability due to the relocalization of junctional proteins, *E. coli* triggers the onset of diarrhea [55]. In chronic cases, the event can lead to EMT. Studies reported that diffusely adherent *E. coli* (DAEC) could infect intestinal epithelial cells and promote EMT by activating MAPK and PI3K pathways (Figure 1) [56,57,58]. In addition, the bacteria will stimulate the overexpression of HIF-1α protein, accentuating loss of E-cadherin and cytokeratin 18 and upregulation of fibronectin, signifying a possible role of *E. coli* in EMT [59].

In contrast, several bacterial products, such as lipopolysaccharide (LPS), flagellin, and muramyl dipeptides (MDP), are extensively studied. LPS, a vital part of the outer membrane of Gram-negative bacteria, is an endotoxin that binds to TLR4 [60]. Although earlier studies reported that LPS-induced EMT is very scarce, Zhao et al. demonstrated that LPS reduced the expression of the epithelial biomarker E-cadherin in intrahepatic biliary epithelial cells [61]. In contrast, the expression of mesenchymal markers S100A4 and α-SMA was enhanced [61]. More importantly, this investigation reported that LPS leads to overexpression of TGFβ-1 [61], an important inducer of EMT via Smad 2/3 [62]. Silencing of Smad 2/3 expression in these cells triggered E-cadherin expression and inhibited S100A and α-SMA deregulation, indicating that LPS induced EMT via the TGFβ1/Smad2/3 pathway [61]. Similar to LPS, flagellin and MDP are also found to trigger EMT. Both flagellin and MDP trigger the NFκB and MAPK signaling pathways [63,64]. In addition, flagellin stimulates the production of TGF-β and TGFβ1, which are known inducers of EMT [65]. Similarly, MDP also induces the expression patterns of genes responsible for invasive cell growth in intestinal epithelial cells and EMT [66].

## 4. Microbiota-Enhanced Carcinogenesis via Epithelial–Mesenchymal Transition

Dysbiosis is associated with host inflammatory responses and EMT in various sites favoring cancer progression. In cancer cells, EMT activation is related to the presence of altered infiltrating tumor-associated macrophages (TAMs), which produce soluble growth factors and inflammatory cytokines and promote extracellular matrix remodeling, angiogenesis, immunosuppression, and cancer cell invasion [67]. In addition, several studies reported that cancer microbiota initiates EMT and tumorigenesis via metabolic reprogramming (Figure 1) [68,69,70]. In the following subsections, we will focus on the presence of microbial colonies in different anatomical sites and their underlying signaling mechanisms responsible for triggering EMT leading to cancer progression.

### 4.1. Respiratory Tract Microbiota

The nasal epithelium is predominated by bacteroidetes, firmicutes, proteobacteria, and actinobacteria [71]. However, a wide variation in the microbial composition has been reported based on several factors, including humid environment, temperature, and localization within the respiratory tract [72]. The nasal microbiota can alter the expression and functions of regulators of the olfactory signaling transduction pathways [71], in addition to the onset of allergic rhinitis and chronic rhinosinusitis [72]. Microbes act as epithelial barriers in the nasal cavity and can promote tissue-remodeling [73]. During microbial infections, the mucociliary clearance is altered, and nasal microbiota is not removed from the airways; thus, they attach to the mucosal surface, form colonies, and produce soluble virulence-associated factors [74]. Ziesemer et al. reported that alpha-hemolysin, a cytotoxic agent released by *Staphylococcus aureus* (*S. aureus*) in human airway epithelial cells, enhanced actin filament remodeling due to disruption of cell-to-cell contact and the focal adhesions leading to the augmented penetrability of the epithelial layer [75]. Moreover, *S. aureus* is involved in nasal polyposis pathogenesis [76]; nasal polyps lack expression of E-cadherin and occluding, while TGFβ and vimentin are overexpressed compared with healthy nasal mucosa [77], indicating a role of *S. aureus* in EMT.

On the other hand, the lung is primarily composed of Bacteroidetes and Firmicutes [78,79]. During respiratory diseases, mucus production presents suitable environmental and nutrient conditions for the microbes to thrive; hence, the microbial composition is altered [80]. This altered microbial composition promotes genotoxic and virulent effects, leading to deregulated metabolism, inflammation, and immune response, features of lung cancer development [80]. A recent study by Jin and colleagues [81] used lung adenocarcinoma mouse models with *Kras* mutations and *p53* deletion to study microbiota-induced inflammation in different myeloid cells. The study reported that in adenoviral (*Sftpc-Cre*) infected mice, the local microbiota activated myeloid cells (neutrophils) to enhance the production of IL-1*β*, IL-23, and *γδ* T cells to stimulate inflammation and tumor cell proliferation through IL-17. Thus, germ-free or antibiotic-exposed mice are drastically protected against lung adenocarcinoma compared to adenoviral (*Sftpc-Cre*) infected mice [81].

Previous studies have investigated the role of gut microbiota in extra-gastrointestinal tumors [33,35], including lung cancer [34,36,37]. Recently, *Enterococcus* and *Bifidobacterium* were associated with the onset of lung cancer and, therefore, suggested as a potential diagnostic biomarker in lung cancer [82]. On the other hand, differential expression of gut microbiota was also observed in lung cancer; the expressions of *Escherichia-Shigella*, *Enterobacter, Dialister*, *Kluyvera*, and *Faecalibacterium* were reduced in lung cancer patients, while *Veillonella*, *Fusobacterium*, and *Bacteroides* were augmented in comparison with healthy individuals [37]. Moreover, non-small-cell lung cancer (NSCLC) patients had higher levels of gut bacteria when compared with healthy controls [83]. However, on the contrary, downregulated levels of gut butyrate-producing bacteria (*Clostridium leptum*, *Faecalibacterium prausnitzii*, *Ruminococcus*, and *Clostridial* cluster I spp.) were recently reported in NSCLC patients [84]. Liu et al. [85] carried out 16S ribosomal RNA (rRNA) gene amplicon sequencing in 30 lung cancer patients compared with 16 healthy individuals. They reported that gut microbiota dysbiosis in lung cancer correlates with altered metabolic and immunologic functions involved in the development and progression of lung cancer.

Similarly, a recent study by Zheng and colleagues utilized the 16S rRNA gene sequencing analysis and revealed the microbiota spectrum of lung cancer patients [86]. The study further reported a potential gut microbial signature for the prediction of early-stage lung cancer [86]. Another recent investigation demonstrated that prebiotics and probiotics have a latent protective effect on lung carcinogenesis [87]. Although studies have reported altered gut microbiome as a potential diagnostic and prognostic marker [88], further studies are warranted to examine the underlying mechanisms of the gut microbiome in the onset and progression of lung cancer.

Nonetheless, studies have also indicated an interaction between the gastrointestinal (GI) and respiratory tracts known as the gut–lung axis by altering microbial and immune functions [89] through a complex bidirectional axis involving blood and lymphatic circulation [90,91]. Dysregulation in the gut–lung axis is implicated in pathogen colonization, tissue damage, and the onset of carcinogenesis [92,93]. There are different pathways involved in the role of the gut–lung axis in lung cancer pathogenesis. TLRs on the intestinal epithelial cells surface identify microbial ligands and induce TLR innate-adaptive immunity; immune cell migration triggers the gut mucous membranes [94]. Inflammation is another mechanism involved in gut–lung-axis-induced lung cancer; microbes migrate from the GI tract to the bloodstream via the mucosal barrier and induce lung inflammation, further augmenting the innate systemic response [95,96,97]. In addition, secondary metabolites produced from bile acids by gut bacteria and alteration of the gut microbiota cause DNA damage, produce toxins, and initiate cancer development; deregulated metabolism triggers toxic metabolite formation in the lungs and contributes to the development of lung cancer [98,99,100]. With the potential role of the gut–lung axis in lung cancer pathogenesis, the possibility of its manipulation for developing biological therapeutic agents needs to be studied further. 

### 4.2. Gastrointestinal (GI) Tract Microbiota

Recently, it has been reported that microbial pathogens, especially intestinal microorganisms, play an essential role in carcinogenesis; intestinal dysbiosis can induce immune response triggering chronic inflammation and, in adverse conditions, leading to cancer progression [101]. 

Oral cancer arises from the oral mucosa, and approximately 15% of the cases are attributed to oral microbial dysbiosis [102]. The oral cavity is inhabited by various microbial species, including *Porphyromonas gingivalis* (*P. gingivalis*), *F. nucleatum*, *Streptococci*, *Peptostreptococci*, and *Prevotella* [103]. Dysbiosis of the oral microbiome alters the immune response resulting in an increased risk of the onset of periodontal diseases and oral squamous cell carcinoma (OSCC) [104,105,106]. Chronic infection in oral cells by *P. gingivalis* induces the expression of CD44 and CD133, which activate matrixins (MMPs-1 and -10) along with Slug, Snail, and Zeb1 leading to EMT [107,108]. This process of chronic-infection-induced EMT in the oral cavity results in oral cells developing invasive and migrative properties [107,108].

The role of microbiota has been assessed in esophageal cancer; in comparison to normal esophagus tissue, reduced microbial diversity is reported in Barrett’s esophagus, esophageal adenocarcinoma (EAC), and esophageal squamous cell carcinoma (ESCC) [109,110,111,112,113,114,115,116,117,118]. In EAC, *Akkermansia* and Gram-negative bacteria, *Lactobacilli*, *Prevotella*, *Leptotrichia*, and *Enterobacteriaceae* are augmented with loss of *Streptococci* [109,114,119]; in ESCC, *Streptococci*, *Fusobacteria*, *Veillonella*, and *P. gingivalis* are abundant with reduced *Lautropia*, *Bulleidia*, *Catonella*, *Corynebacterium*, *Moryella*, *Peptococcus*, *Treponema*, and *Cardiobacterium* [111,115,116,120]. An in vivo study using a xenograft model reported microbial pathogens to play a role in increased uptake of metabolic glucose in addition to EMT in the esophagus [121]. Moreover, dietary intake is reported to affect the microbial composition in esophageal cancer pathogenesis. Kaakoush and colleagues performed an in vivo study using Sprague Dawley rats; the rats were given an obesogenic diet and had an altered esophageal microbiota associated with chronic gastrointestinal diseases compared with normal diet-fed rats [122]. Another recent in vivo study involved transgenic mice (L2-IL1B mice) fed a high-fat diet; the study reported dysbiosis of the esophageal and gut microbiota resulting in inflammation and development of esophageal tumors in comparison with mice fed a normal diet [123]. Moreover, Riboflavin, a vitamin B2 supplement, impacts the balance between gut microbiota and esophageal mucosal integrity [124]. In vivo studies reported that riboflavin deficiency alters the gut microbiota and leads to esophageal epithelial atrophy [125,126]. The role of *H. pylori* in esophageal cancer is conflicting. Although the reduced *H. pylori* incidence is associated with an increased risk of EAC, there was no significant association between *H. pylori* infection and ESCC [127,128,129]. However, one study reported that *H. pylori* infection is associated with ESCC in the non-Asian population; in the Asian population, it showed a converse relation [128]. On the contrary, studies in the US and Swedish populations failed to find an association between *H. pylori* infection and EAC incidence [130,131]. These studies indicate a need to investigate the role of *H. pylori* in the onset and development of esophageal cancer.

However, *H. pylori* is the most common cause of gastric cancer and is classified as a class I carcinogen involved in the onset of gastric cancer pathogenesis by inducing inflammation and alteration of the gastric mucosal integrity [132,133,134,135,136]. Human gastric microbiota profiling revealed differential microbiota profiles between chronic gastritis, metaplasia, and gastric cancer, indicating that dysbiosis is associated with cancer progression [132,137,138,139,140,141,142,143]. Gastric colonization with *H. pylori* and *Clostridium*, *Lactobacillus*, and *Bacteroides* enhance inflammation with upregulated IL-11 expression and oncogenic genes, *Ptger4*, and *Tgf-β*, plausibly regulated by the yes-associated protein 1 (YAP1) [123,144]. Gastric microbiota analysis using 16S rRNA gene profiling demonstrated a distinct dysbiotic microbial community with plausible genotoxicity in gastric cancer in comparison to chronic gastritis [132]. In addition to the abundance of *H. pylori* in gastric cancer, oral-associated bacteria have also been found in patients with gastric cancer [136]. Several other investigations also reported the loss of *H. pylori* in gastric cancer in lieu of the dominant presence of *Clostridium*, *Enterococci*, *Fusobacterium*, *Veillonella*, *Leptotrichia, Staphylococci*, and *Lactobacillus* species [141,145]. Similarly, another study reported the presence of *F. nucleatum* to correlate with an overall worse prognosis in Laurens’s diffuse-type gastric cancer [146]. Lately, in vivo studies revealed that a high-fat diet in mice stimulates gastric dysbiosis and the enhanced presence of *Lactobacilli*, intestinal metaplasia, STAT3, and accumulation of β-catenin; these changes provide a protumorigenic gastric microenvironment leading to the onset and development of gastric cancer [147,148]. 

Contrary to gastric cancer, the role of *H. pylori* in colorectal cancer (CRC) pathogenesis is unclear. Dysbiosis of the gut microbiota is reported in tissues of CRC when compared with normal tissue [23,24,25,26,27,28,29,30,31,32]. Sears and Pardoll suggested the “alpha-bug” (enterotoxigenic *Bacteroides fragilis*) hypothesis for colorectal cancer, where they found that oncogenic microorganisms can modify the mucosal immune response and colonic bacterial community to promote colorectal cancer [149]. On the other hand, Tjalsma and colleagues [150] proposed another model for colorectal cancer, known as the “driver passenger”, where they explained that tumors induced by microbes (driver) are subsequently replaced by other symbiotic microbes (passengers) and can alter the local infectious environment, further promoting tumorigenesis. Different gut microbiota species are found in different stages of CRC progression; Gram-positive bacteria (*Firmicutes* and *Actinobacteriaphyla*) are dominant in premalignant adenomas, whereas Gram-negative bacteria (*Enterobacteriaceae*, *Proteobacteria*, *Burkholderiales*, and *Sutterellai*) are dominant in CRC [151]. Furthermore, the microbe *Oscillospira* is lost during the transition from advanced adenoma to early CRC [30]. One commonly detected microbial pathogen in CRC is *F. nucleatum*, which correlates with an elevated risk of CRC recurrence and chemoresistance [45,152,153]. *F. nucleatum* adheres to the colonic mucosa and interacts with Fap2 and integrin α_2_/β_1_ promoting cell proliferation and triggering the NF-κB pathway (Figure 1), in addition to the inhibition of natural killer cell response and accumulation of myeloid cells. These events alter the tumor microenvironment leading to microbial metastatic spread [45,154]. Furthermore, *E. coli* is also associated with the development of colon cancer; *E. coli* regulates the production of colibactin, a genotoxic *E. coli* strain resulting in DNA double-strand breaks, gut microbiota dysbiosis, stimulation of the NF-κB and Wnt/β-catenin pathways, as well as inflammation of the colonic mucosa, further stimulating cell proliferation [155,156]. In addition to these microbes, Fragilysin is another microbe present in the gut [157]. Fragilysin attaches to the epithelial receptors of the colon and initiates the NF-κB pathway, thus leading to an increase in colon cell growth, proliferation, and DNA damage [158,159]. On the other hand, Fragilysin also triggers cell proliferation and c-MYC activation by deregulating the Wnt/β-catenin signaling pathway via E-cadherin cleavage [158,159,160].

Likewise, a recent report indicates that oral and gut microbiota dysbiosis enhance bacterial invasion, which correlates with pancreatic cancer incidence [161]; however, studies are scarce on this particular topic. At the same time, other investigations reported that *P. gingivalis* in the oral cavity increased the risk of the onset of pancreatic ductal adenocarcinoma and cancer [162,163]. However, the role of *H. pylori* is contradictory in pancreatic cancer; while Wei et al. [164] suggested *H. pylori* as a risk factor for the development of pancreatic cancer, another study failed to detect *H. pylori* in pancreatic tissue or fluid by PCR [165]. It is also evidenced that *H. pylori* secretes cytotoxins and vacuolins, and induces chronic inflammation and DNA damage, leading to pancreatic carcinogenesis [165,166]. Furthermore, 16s rRNA gene sequencing in pancreatic ductal adenocarcinoma identified 13 different microbe phyla, of which the most abundant were *Proteobacteria*, followed by *Bacteroids* and *Firmicutes* [167]. Commonly, duodenal or biliary bacterial reflux promotes translocation and colonization of the gut microbiota in the pancreas [167], enhancing the development and progression of pancreatic cancer [168].

### 4.3. Female Reproductive Tract Microbiota

The cervicovaginal tract comprises a diversified and complex microbial community named cervicovaginal microbiome (CVM), regulating different physiological disorders [169,170]. Although the CVM is composed of different microbe communities, it is highly dominated by the genus *Lactobacillus* (*Lactobacillus crispatus*, *Lactobacillus iners*, *Lactobacillus gasseri*, or *Lactobacillus jensenii*) [171,172]. In addition to maintaining tissue homeostasis [173] and a local pH lesser than 4.5 [174], lactobacilli adhere to epithelial cells by forming microcolonies and serve as a barrier to protect the genital environment from infectious pathogens [175], counteracting bacterial vaginosis, yeast infections, and sexually transmitted diseases (STDs) [176,177]. The imbalance of the CVM triggers abnormal cell proliferation, chronic inflammation, genome instability, STDs, premature births, and cancers of the vaginal tract [40,178,179,180]. Enhanced vaginal dysbiosis induces proinflammatory cytokines and chemokines production, followed by an inflammatory response [181] and dysregulation of the immune response favoring a tumor-promoting microenvironment [182,183]. The presence of *Atopobium vaginae* and *Porphyromonas* sp. in the reproductive tract, along with an increased vaginal pH (>4.5), correlated with the onset of endometrial cancer [184]. On the other hand, in cervical cancer, Laniewski et al. [39] reported that a low abundance of lactobacilli is associated with increased vaginal pH and enhanced secretion of various inflammatory cytokines, including interleukins (IL-2, IL-4, and IL-36*γ*), MIP-1*β*, IP-10, Flt-3L, and sCD40L. A study by Mitra and colleagues [185] reported high bacterial variation and loss of lactobacilli to be associated with cervical intraepithelial neoplasia (CIN) progression and cytological lesion severity. In addition, several studies have linked vaginal dysbiosis with human papillomavirus (HPV) infection in different grades of CIN and cervical cancer [185,186,187,188]. A study by Kwasniewski [189] reported dysbiosis of vaginal microbiota to induce the development of HPV-induced cervical cancer, indicating a role of vaginal microbiota in regulating viral persistence. Other studies also reported an association of reduced lactobacilli with an increased risk of HPV infection and bacterial vaginosis [186,190]. Lactobacilli reduce microbiome composition, triggering inflammation that can stimulate the expression of high-risk HPV oncogenes (E6 and E7) and malignant cell proliferation [191]. Studies also reported differential expression of microbiota in ovarian cancer tissues compared with normal tissues. Chronic infection with *Proteobacteria* and *Firmicutes* induces an inflammatory immune response leading to the onset and progression of ovarian carcinogenesis [38,41].

Since the gut microbiota shares approximately 30% of bacterial species, including *Firmicutes, Bacteroidetes*, *Proteobacteria, Actinobacteria*, and *Fusobacteria* [191], and regulates circulating estrogen (estrobolome), it is suggested that there is a crosslink between the gut/estrobolome and related risk of vaginal diseases including malignancies [191,192,193,194]. The gut microbiome may be regarded as a reservoir for vaginal microbes. The Group B streptococcus is present in the gut; however, if present in the vagina of pregnant women, it can induce premature delivery [195]. Enhanced levels of lactobacilli in the vagina reduce bacterial vaginosis [196]. However, the intake of oral probiotics was found to inhibit bacterial vaginosis, indicating an influence on the gut microbiome in the vagina [197]. In addition, *Lactobacillus*, *Bacteroides*, *Bifidobacterium*, and *Akkermansia* are associated with enhanced levels of short-chain fatty acids (SCFA) [198]; a differential role of SCFA has been shown between the gut and the vagina [191]. In the gut, SCFAs have anti-inflammatory characteristics and regulate the intestinal epithelial barrier [198]; whereas, in the vagina, SCFAs’ expression might be linked with several proinflammatory biomarkers [199]. In ovarian cancer, Xu et al. [200] demonstrated that intestinal dysbiosis activates tumor-associated macrophages and increases circulating levels of proinflammatory cytokines (IL-6 and TNF-α), promoting the onset of EMT.

Nevertheless, vaginal pathogens inducing diseases of the gut are still inconclusive. While studies reported delivery via the vagina or cesarean section to protect against asthma and gastroenteritis [201,202], another study did not find any association between the mode of delivery and respiratory or gut diseases [203]. While the gut microbiome is contemplated as one of the vital regulators of circulating estrogens, studies supporting the role of estrogen-related signaling and high-risk HPV-induced cancer are nascent and warrant further research [192,193,194]. Table 1 summarizes the roles of various gut microbiota in the onset of some common cancers.

## 5. Microbiome-Based Therapies (Biotherapy)

Gut microbiota in EMT-induced carcinogenesis is also involved in response to cancer therapy and toxicities [219,220]. The gut microbiota dysbiosis can modify both the systemic immune system and the response to chemotherapeutic agents [221,222]. However, cancer therapeutic drugs and antibiotics administration during the surgical or chemotherapeutic intervention can alter the gut microbiota. Moreover, chemotherapy and radiotherapy induce significant gut dysbiosis by destroying intestinal or colonic mucosa and altering several metabolic pathways leading to the risk of colitis [223,224,225].

To overcome these challenges, studies have focused on restoring the gut microbiota and helped pave the way for therapeutic strategies. For instance, fecal microbiota transplantation (FMT) was primarily used to treat *Clostridioides difficile* infection (CDI) by retention enemas and became common practice over the last decade [226,227]. FMT is administered through several ways, including infusion via nasogastric tube, oral capsules colonoscopy, and enema [228]; similar response rates were achieved for both oral administration and colonoscopy [229]. FMT is emerging as a candidate therapeutic option for treating several gut dysbiotic nonmalignant diseases, including irritable bowel syndrome, inflammatory bowel disease, multidrug-resistant diseases, metabolic syndrome, diabetes, nonalcoholic fatty liver disease, neuropsychiatric disorders, and autoimmune diseases [230,231,232,233]. However, although there are clinical trials observing the use of FMT against cancer in clinical practice, this still lies nascent [234,235,236].

On the other hand, probiotics involve the intake of bacteria or a combination of live organisms via supplements to maintain the normal microflora in the body [237]. Research has explored several commercially available probiotics in clinical trials, especially in CRC tumorigenesis. Interestingly, such studies demonstrated the efficacy of probiotic VSL#3 in CRC [238,239]; contrarily, another investigation reported that VSL#3 alters the mucosal microbial composition and enhances tumor growth [240]. In addition, although the effect of probiotic administration has been examined in several clinical trials in cancer patients, the studies majorly focused on the analysis of microbe dysbiosis [241,242,243,244,245]. Hence, more studies are required to assess the differential outcome of probiotics against cancer.

As previously stated, diet plays a role in gut microbiota composition and their metabolomic and transcriptomic profiles [147,246,247]. Numerous reports have indicated diet intake as a potential anticancer intervention [246,248,249]. On the other hand, prebiotics and postbiotics can also alter gut microbiota. Substances, including fructans, induce the growth of certain bacteria and modify SCFA levels within the gut; fructans were found to increase the efficacy of chemo- and radiotherapeutic agents in murine models [250]. In humans, use of postbiotics was studied against CRC and it was found that intake of butyrogenesis from high-fat-diet foods suppressed CRC carcinogenesis [251].

Finally, it is known that the use of antibiotics is associated with significant alteration in gut microbiota and worse clinical outcomes [252]. For example, patients with NSCLC demonstrated poor prognoses when given antibiotics before and after the start of treatment with immune checkpoint blockade [253]. Similarly, when administered anti-Gram-positive antibiotics, chronic lymphocytic leukemia patients had poor overall survival and response rates and earlier disease progression [253]. However, it might be useful to develop targeted antibiotics and bacteriophages to target the microbiota efficiently and improve therapeutic response selectively. In contrast, bacteriophages are the most significant and distinct members of the gut virobiota and have demonstrated efficiency in structuring the gut microbiota and targeting specific bacterial colonies [254,255]. Although these studies highlight the critical role of gut microbiota and biotherapy in the management of certain diseases, including cancer, additional studies are warranted to understand the underlying mechanisms and their plausible impact on the normal flora and immune system.

## 6. Conclusions

This review presents a concise outlook on the role of dysbiotic microbiota in EMT by altering transcription factors and deregulating signaling pathways, mainly STAT3, Wnt/β-catenin, and NF-κB. Although the role of microbes is well-defined in health and disease, their function in enhancing cancer progression via EMT is still nascent. Microbes inducing fibrin production or cancer have been implicated in EMT. Hence, understanding and unraveling the impact of the microbiota in inducing EMT and, therefore, cancer progression can help develop novel therapeutic regimens and biotherapies for human diseases, including cancers.

## Figures and Tables

**Table 1:** Overview of the studies exploring the gut-microbiota-associated human cancers.

Study	Detection Method	Bacterium Species	Expression Levels
Colorectal Cancer			
Boehm et al. (2020) [146]	Probe-based quantitative PCR	*Fusobacterium nucleatum*	Upregulated
Mori et al. (2018) [151]	16S rRNA gene sequencing	*Sutterella* and *Escherichia/Shigella*	Upregulated
Yu et al. (2017) [153]	Quantitative PCR	*Fusobacterium nucleatum*	Upregulated
Mima et al. (2015) [204]	Molecular pathological epidemiology database	*Fusobacterium nucleatum*	Upregulated
Mira-Pascual et al. (2015) [205]	16S rRNA gene pyrosequencing and quantitative PCR	*Methanobacteriales*, *Methanobrevibacterium*, *Fusobacterium nucleatum*, *Enterobacteriaceae*, *Akkermansia muciniphila*, and *Blautia coccoides*	Upregulated
		*Bifidobacterium, Faecalibacterium prausnitzii*, and *Lactobacillus*	Downregulated
Tahara et al. (2014) [206]	Quantitative real-time PCR	*Fusobacterium nucleatum* and *pan-fusobacterium*	Upregulated
Zackular et al. (2014) [32]	16S rRNA gene sequencing	*Ruminococcaceae*, *Clostridium*, *Pseudomonas*, and *Porphyromonadaceae*	Upregulated
Bonnet et al. (2014) [155]	PCR	*Escherichia coli*	Upregulated
Nugent et al. (2014) [207]	Quantitative real-time PCR	*Bifidobacterium*, *Eubacteria*, *Escherichia coli*, *Clostridium*, and *Bacteroides*	Upregulated
Wu et al. (2013) [208]	Pyrosequencing of the 16S rRNA gene V3 region	*Bacteroids*, *Fusobacterium*, and *Campylobacter*	Upregulated
		*Faecalibacterium* and *Roseburia*	Downregulated
Warren et al. (2013) [209]	Metatranscriptomic analysis	*Fusobacterium*, *Leptotrichia*, and *Campylobacter*	Upregulated
McCoy et al. (2013) [210]	16S rRNA quantitative PCR and pyrosequencing	*Fusobacterium*	Upregulated
Brim et al. (2013) [211]	Human intestinal Tract Chip (HITChip) and 16S rRNA gene barcoded 454 pyrosequencing	*Bacteroidetes* and *Firmicutes*	Upregulated
Castellarin et al. (2012) [152]	Quantitative PCR	*Fusobacterium nucleatum*	Upregulated
Sanapareddy et al. (2012) [212]	454 titanium pyrosequencing of the V1–V2 region of the 16S rRNA gene	*Firmicutes*, *Bacteroidetes*, *Pseudomonas*, *Helicobacter*, *Actinobacteria*, *Lactobacillus*, *Acinetobacter*, and *Proteobacteria*	Upregulated
Marchesi et al. (2011) [213]	Deep rRNA sequencing	*Roseburia*, *Fusobacterium*, and *Faecalibacterium*	Upregulated
		*Citrobacter*, *Shigella*, *Cronobacter*, *Kluyvera*, *Serratia*, and *Salmonella* spp.	Downregulated
Shen et al. (2010) [214]	Terminal restriction fragment length polymorphism, clone sequencing and fluorescent in situ hybridization analysis of the 16S rRNA genes	*Dorea* spp. and *Faecalibacterium* spp.	Upregulated
Esophageal Cancer			
Nie et al. (2014) [128]	Meta-analysis	*Helicobacter pylori*	Downregulated
Chow et al. (1998) [127]	Antigen-specific ELISA	*Helicobacter pylori*	Downregulated
Gastric Cancer			
Boehm et al. (2020) [146]	Probe-based quantitative PCR	*Fusobacterium nucleatum*	Upregulated
Hansen et al. (2020) [134]	18S rDNA sequencing	*Malassezia*	Upregulated
Hsieh et al. (2018) [145]	16S ribosomal DNA analysis	*Fusobacterium* and *Clostridium*	Upregulated
		*Helicobacter pylori*	Downregulated
Ferriera et al. (2018) [132]	16S rRNA next-generation sequencing	*Helicobacter pylori*	Downregulated
Yu et al. (2017) [136]	16S rRNA gene sequencing	*Helicobacter pylori*	Upregulated
Sohn et al. (2017) [140]	Bar-coded 454 pyrosequencing of the 16S rRNA gene	*Streptococcus pseudopneumoniae, S. parasanguinis*, and *S. oralis*	Upregulated
Aviles-Jimenez et al. (2014) [139]	Microarray G3 PhyloChip analysis	*Pseudomonas*, *Lactobacillus coleohominis*, and *Lachnospiraceae*	Upregulated
		*Porphyromonas*, *TM7*, *Neisseria*, and *Streptococcus sinensis*	Downregulated
Dicksved et al. (2009) [215]	Terminal restriction fragment length polymorphism analysis in combination with 16S rRNA gene cloning and sequencing	*Streptococcus*, *Lactobacillus*, *Veillonella*, and *Prevotella*	Upregulated
Chow et al. (1998) [127]	Antigen-specific ELISA	*Helicobacter pylori*	Downregulated
Lung Cancer			
Sobhani et al. (2011) [216]	Quantitative PCR and pyrosequencing	*Helicobacter pylori*	Downregulated
		*Bifidobacterium*, *Faecalibacterium*, *Streptococcus*, and *Veillonella*	Downregulated
Gui et al. (2020) [84]	Quantitative PCR	*Faecalibacterium prausnitzii*, *Clostridium leptum*, *Ruminococcus* spp., *Clostridial cluster I*, *Clostridial cluster XIVa*, and *Roseburia* spp.	Downregulated
Zhuang at el. (2019) [82]	16S rRNA next-generation sequencing	*Enterococcus*	Upregulated
		*Bifidobacterium*	Downregulated
Liu et al. (2019) [137]	16S rRNA gene amplicon sequencing	*Fusobacteria*, *Prevotella* *Proteobacteria*, *Streptococcus*, *Verrucomicrobia*, and *Veillonella*	Upregulated
		*Bacteroidetes*, *Firmicutes*, and *Actinobacteria*	Downregulated
Zhang et al. (2018) [37]	16S rRNA gene sequencing	*Bacteroides*, *Veillonella*, and *Fusobacterium*	Upregulated
		*Escherichia-Shigella*, *Kluyvera*, *Fecalibacterium*, *Enterobacter*, and *Dialister*	Downregulated
Apostolou et al. (2011) [217]	Reverse-transcription polymerase chain reaction	*Staphylococcus epidermidis*, *Streptococcus mitis*, and *Bacillus strains*	Upregulated
Pancreatic Ductal Adenocarcinoma			
Jesnowski et al. (2010) [165]	Nested PCR	*Helicobacter pylori*	No expression
Ovarian Cancer			
Chan et al. (1996) [218]	Combined PCR-ELISA Assay	*Mycoplasma*	Upregulated

## Data Availability

Not applicable.

## Abbreviations

α-SMA	Alpha smooth muscle actin
β-catenin	Beta-catenin
γδ T cells	Gamma delta T cells
CagA	Cytotoxin-associated gene A
CIN	Cervical intraepithelial neoplasia
c-MYC	Cellular myelocytomatosis
CRC	Colorectal cancer
CVM	Cervicovaginal microbiome
DAEC	Diffusely adherent
EAC	Esophageal adenocarcinoma
E-cadherin	Epithelial cadherin
ECM	Extracellular matrix
*E. coli*	*Escherichia coli*
EMT	Epithelial mesenchymal transition
ERK	Extracellular-signal-regulated kinase
ESCC	Esophageal squamous cell carcinoma
*F. nucleatum*	*Fusobacterium nucleatum*
Flt-3L	FMS-like tyrosine kinase 3 ligand
GI	Gastrointestinal
HIF-1α	Hypoxia-inducible factor 1-alpha
*H. pylori*	*Helicobacter pylori*
HMP	Human Microbiome Project
HPV	Human papillomavirus
IL	Interleukin
IP-10	Interferon gamma-induced protein 10
Kras	Kirsten rat sarcoma viral oncogene homolog
LPS	Lipopolysaccharide
MAPK	Mitogen-activated protein kinase
MDP	Muramyl dipeptides
MIP	Macrophage Inflammatory Proteins
MMPs	Matrix metalloproteases
N-cadherin	Neural cadherin
NF-κB	Nuclear factor kappa light chain enhancer of activated B cells
NSCLC	Non-small-cell lung cancer
OSCC	Oral squamous cell carcinoma
PCR	Polymerase chain reaction
*P. gingivalis*	*Porphyromonas gingivalis*
p53	Tumor protein 53
PI3K	Phosphatidylinositol 3-kinase
Ptger4	Prostaglandin E Receptor 4
Ras	Rat sarcoma virus
ROS	Reactive oxygen species
rRNA	Ribosomal RNA
S100A	S100 Calcium Binding Protein A1
*S. aureus*	*Staphylococcus aureus*
SCFA	Short chain fatty acids
SMAD	Suppressor of Mothers against Decapentaplegic
STAT	Signal transducer and activator of transcription
STDs	Sexually transmitted diseases
TAMs	Tumor-associated macrophages
TGFβ	Transforming growth factor β
TILs	Tumor-infiltrating lymphocytes
TLR	Toll-like receptor
TNF	Tumor necrosis factor
VacA	Vacuolating toxin A
Wnt	Wingless
YAP1	Yes-associated protein 1
Zeb1	Zinc Finger E-Box Binding Homeobox 1.
